# Rational Design of a Water‐Storable Hierarchical Architecture Decorated with Amorphous Barium Oxide and Nickel Nanoparticles as a Solid Oxide Fuel Cell Anode with Excellent Sulfur Tolerance

**DOI:** 10.1002/advs.201700337

**Published:** 2017-09-15

**Authors:** Yufei Song, Wei Wang, Lei Ge, Xiaomin Xu, Zhenbao Zhang, Paulo Sérgio Barros Julião, Wei Zhou, Zongping Shao

**Affiliations:** ^1^ State Key Laboratory of Materials‐Oriented Chemical Engineering College of Chemical Engineering Jiangsu National Synergetic Innovation Center for Advanced Materials (SICAM) Nanjing Tech University No. 5 Xin Mofan Road Nanjing 210009 P. R. China; ^2^ Department of Chemical Engineering Curtin University Perth Western Australia 6845 Australia; ^3^ Center for Future Materials University of Southern Queensland Springfield Central Queensland 4300 Australia; ^4^ State Key Laboratory of Materials‐Oriented Chemical Engineering School of Energy Science and Engineering Jiangsu National Synergetic Innovation Center for Advanced Materials (SICAM) Nanjing Tech University No. 5 Xin Mofan Road Nanjing 210009 P. R. China

**Keywords:** anode, energy conversion, solid oxide fuel cells, sulfur tolerance, water‐storable material

## Abstract

Solid oxide fuel cells (SOFCs), which can directly convert chemical energy stored in fuels into electric power, represent a useful technology for a more sustainable future. They are particularly attractive given that they can be easily integrated into the currently available fossil fuel infrastructure to realize an ideal clean energy system. However, the widespread use of the SOFC technology is hindered by sulfur poisoning at the anode caused by the sulfur impurities in fossil fuels. Therefore, improving the sulfur tolerance of the anode is critical for developing SOFCs for use with fossil fuels. Herein, a novel, highly active, sulfur‐tolerant anode for intermediate‐temperature SOFCs is prepared via a facile impregnation and limited reaction protocol. During synthesis, Ni nanoparticles, water‐storable BaZr_0.4_Ce_0.4_Y_0.2_O_3−_ *_δ_* (BZCY) perovskite, and amorphous BaO are formed in situ and deposited on the surface of a Sm_0.2_Ce_0.8_O_1.9_ (SDC) scaffold. More specifically, a porous SDC scaffold is impregnated with a well‐designed proton‐conducting perovskite oxide liquid precursor with the nominal composition of Ba(Zr_0.4_Ce_0.4_Y_0.2_)_0.8_Ni_0.2_O_3−_ *_δ_* (BZCYN), calcined and reduced in hydrogen. The as‐synthesized hierarchical architecture exhibits high H_2_ electro‐oxidation activity, excellent operational stability, superior sulfur tolerance, and good thermal cyclability. This work demonstrates the potential of combining nanocatalysts and water‐storable materials in advanced electrocatalysts for SOFCs.

A useful strategy for a cleaner, more sustainable future is to improve the energy conversion efficiency of fossil fuels with minimized emissions. Fuel cell technology enables chemical energy stored in the fuels to be converted to electric power via an electrochemical route that is not limited by the Carnot cycle, thus ensuring high efficiency.^1^, ^2^, ^3^ Furthermore, the exhaust gas is not diluted by N_2_, which would make subsequent CO_2_ sequestration easier and therefore results in a decrease in the greenhouse gas emissions. Among the various types of fuel cells, solid oxide fuel cells (SOFCs) are particularly attractive because they can be easily integrated into currently available fossil fuel infrastructure to obtain an ideal clean energy system.^4^, ^5^, ^6^, ^7^ To realize their widespread use, however, some important issues must be resolved. Because fossil fuels contain sulfur impurities, even after the purification, the anode must exhibit high sulfur tolerance. H_2_S concentrations as low as a few ppm can cause a dramatic decrease in the performance of cells with conventional Ni‐based cermet anodes.^8^ Due to the low activation energy of H_2_S dissociation at these anodes, sulfur atoms are easily generated and block the active sites on the Ni surface, thereby inhibiting the fuel oxidation.^9^ Therefore, improving the anode sulfur tolerance is critical for developing SOFCs for use with fossil fuels.

Over the past decade, much research has been focused on developing new materials and architectures, such as Ni alloys with sulfur‐tolerant elements, oxide electrodes, anode functional layers, unique anode architectures, and nanocatalyst‐modified anode surfaces,^10^, ^11^, ^12^, ^13^, ^14^ for improving the sulfur tolerance of SOFC anodes. However, none of the tested systems meets all the strict requirements for practical SOFC anodes, such as good compatibility with other cell components, high conductivity, high fuel electro‐oxidation/reforming activities and excellent coking/sulfur resistance. For example, the introduction of a sulfur‐tolerant metal decreased the electro‐oxidation activity of the anode, whereas the oxide‐based anodes suffer from poor electronic conductivity and electro‐oxidation activity at low temperatures.

To date, extensive studies have shown that Ni is the preferred electrocatalyst for fuel electro‐oxidation in SOFCs due to its superior activity, conductivity, and thermal compatibility.^15^, ^16^ However, this catalyst must be modified to enable its direct use with fossil fuels. One important modification strategy is to decrease the Ni particle size to nanometer scale to improve the interaction between Ni and substrate.^17^, ^18^, ^19^ Exsolution of Ni‐based nanoparticles from a perovskite lattice under reducing conditions at high temperatures is a promising approach to preparing ultrafine Ni‐based nanoparticles with excellent coking/sulfur tolerance and activity.^18^, ^19^ However, this strategy usually produces a limited number of Ni‐based nanoparticles. Very recently, Du et al. reported the fabrication of an Sr_3_FeMoO_7−_ *_δ_* and SrFe*_x_*Mo_1−_ *_x_*O_3−_ *_δ_* composites decorated with FeNi_3_ alloy nanoparticles by reducing the Sr_2_FeMo_0.65_Ni_0.35_O_6−_ *_δ_* double perovskite oxide at a high temperature, showing favorable activity and high coking resistance.^20^ The number of Ni–Fe alloy nanoparticles deposited on the composite was high due to the conversion‐type reaction used to prepare it. However, the thermal compatibility of this anode with other cell components remains a major concern because the phase transition that occurred during reduction might induce considerable thermal expansion.

Another important strategy for improving the coking/sulfur tolerance of Ni‐based anodes is to increase the gasification rate of deposited carbon/sulfur on the anode.^21^, ^22^, ^23^ In the oxygen‐ion‐conducting SOFCs, water is produced at the anode under current polarization, which can be used to remove the deposited carbon/sulfur on the anode. In a pioneering work, Liu and co‐workers reported that BaO nanoparticles deposited on Ni surface can adsorb water, facilitating the rapid removal of the deposited carbon/sulfur on the anode.^21^ More recently, Shao and co‐workers demonstrated that using a water‐storable proton conductor in a Ni‐based anode resulted in excellent coking/sulfur resistance.^22^, ^23^ However, the operational stability of this anode was unsatisfactory due to the large Ni particle size and poor contact between Ni and the water‐storable phase.^23^

Due to the complexity of the electrode reactions at SOFC anodes, it is difficult to simultaneously achieve high electro‐oxidation activity and good coking/sulfur tolerance by a single strategy; therefore, a combination of various strategies is desirable. Herein, we prepared a novel, highly active, sulfur‐tolerant SOFC anode by a facile impregnation and limited reaction protocol. First, a proton‐conducting perovskite oxide with a nominal composition Ba(Zr_0.4_Ce_0.4_Y_0.2_)_0.8_Ni_0.2_O_3−δ_ (BZCYN) was designed and then introduced into a porous SDC scaffold by impregnation, followed by calcination. Then, a limited reaction was performed by H_2_ treatment at 800 °C to obtain a porous hierarchical architecture consisting of Ni nanoparticles, water‐storable BaZr_0.4_Ce_0.4_Y_0.2_O_3−δ_ (BZCY) perovskite and amorphous BaO deposited on SDC scaffold. The obtained anode exhibited excellent sulfur tolerance, H_2_ electroactivity, durability, and thermal cyclability at intermediate temperatures. This work opens a new avenue for the rational design of SOFC anodes with great potential in practical applications.

The capability of Ni doping into BZCY perovskite lattice by synthesis under oxidizing atmosphere and Ni exsolution from the perovskite lattice under reducing atmosphere was first confirmed by X‐ray diffraction (XRD). As shown in Figure S1 in the Supporting Information, crystalline NiO phase was not detected in the as‐synthesized BZCYN sample. Instead, a pure cubic perovskite structure with a lattice parameter of 4.292 Å, which is slightly smaller than that of Ni‐free BZCY (4.302 Å), was observed. The smaller BZCYN lattice parameter is consistent with the fact that the ionic radius of Ni^2+^ (0.69 Å) is smaller than those of Zr^4+^ (0.72 Å), Ce^4+^ (0.87 Å), Ce^3+^ (1.02 Å), and Y^3+^ (0.90 Å). The decrease in the lattice parameter and absence of NiO crystalline phase strongly indicated that Ni was successfully doped into BZCYN perovskite lattice. After H_2_ treatment, a weak metallic Ni peak appeared, indicating successful Ni exsolution from the perovskite lattice. Moreover, the lattice parameter of the main perovskite phase increased to 4.310 Å, possibly due to Ni exsolution and Ce^4+^ partial reduction (Figure S2, Supporting Information). The successful Ni exsolution from the perovskite lattice after H_2_ treatment was further confirmed by Ni 2p X‐ray photoelectron spectroscopy (XPS) in **Figure** **1**a. Before reduction, the Ni 2p peaks of BZCYN sample were observed at 858.0 and 864.0 eV, indicating the presence of Ni^2+^. After H_2_ treatment, Ni^2+^ peaks disappeared and new Ni^0^ peaks appeared at 856.2 and 863.0 eV. Interestingly, XPS survey spectra show that the Ba atomic percentage of BZCYN surface increased from 40.0 to 47.0 at% after H_2_ treatment (Figure 1b), suggesting that Ni exsolution was accompanied by BaO surface enrichment. Therefore, the following reaction was proposed to occur in H_2_ treatment: Ba(Zr_0.4_Ce_0.4_Y_0.2_)_0.8_Ni_0.2_O_3−_ *_δ_* + 0.2H_2_ → 0.8BaZr_0.4_Ce_0.4_Y_0.2_O_3−_ *_δ_* + 0.2BaO + 0.2Ni + 0.2H_2_O. The absence of BaO crystalline phase peaks in the XRD pattern revealed its amorphous nature. Because the dissolution of free BaO occurs more readily in water than Ba^2+^ leaching from BZCYN perovskite lattice, the Ba^2+^ concentration of the reduced BZCYN soaking water was much higher than that of the as‐synthesized BZCYN soaking water (125 vs 54 ppm), verifying the formation of BaO phase. Although the perovskite structure can tolerate a large A‐site cation deficiency, a B‐site cation deficiency is usually energetically unfavorable.^24^ Ni exsolution from BZCYN during H_2_ treatment resulted in the formation of B‐site cation‐deficient perovskite, which promoted Ba leaching from the perovskite lattice.

**Figure 1:**
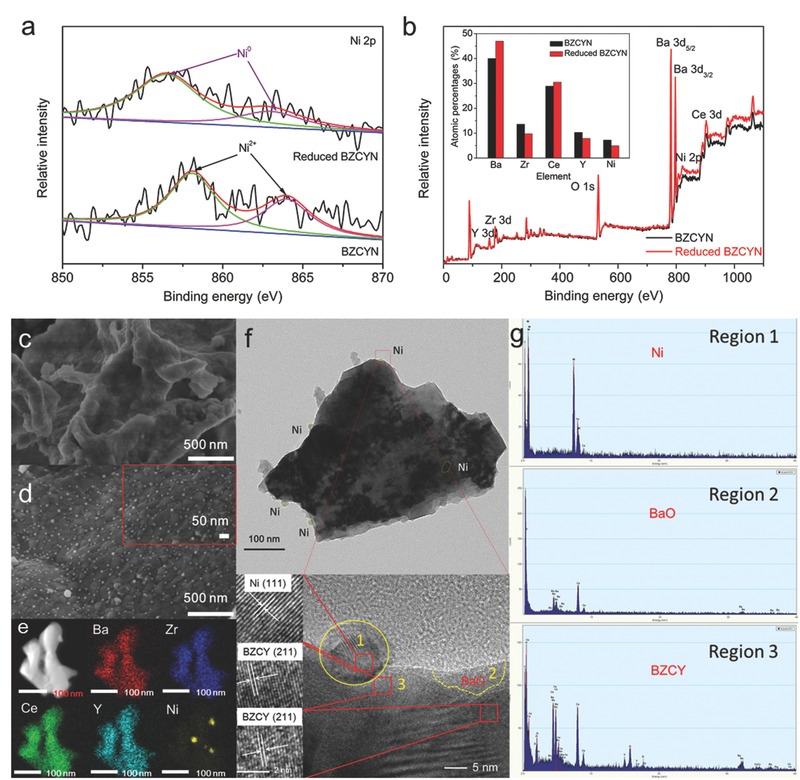
XPS a) Ni 2p and b) survey spectra of as‐synthesized and reduced BZCYN anodes. The Ba, Zr, Ce, Y, and Ni atomic percentages are shown in the inset. SEM images of c) as‐synthesized and d) reduced BZCYN anodes, e) STEM‐EDX results, f) TEM images and g) corresponding EDX results for the reduced BZCYN anode.

The morphologies and phase compositions of the as‐synthesized and reduced BZCYN were examined by scanning electron microscopy (SEM), high‐resolution transmission electron microscopy (HR‐TEM) and scanning transmission electron microscopy (STEM) combined with energy‐dispersive X‐ray (EDX) spectroscopy. The as‐synthesized BZCYN surface was smooth (Figure 1c), whereas many nanoparticles with an average size of ≈25 nm (Figure 1d; Figure S3, Supporting Information) were observed on reduced BZCYN surfaces. Figure 1e shows that the large grains of reduced BZCYN were composed of Ba, Zr, Ce, and Y elements, whereas the nanoparticles were composed of Ni. Figure 1f shows that the reduced BZCYN sample consisted of two components: large, dark particles and lighter nanoparticles on their surfaces. The HR‐TEM and EDX results further confirmed that the nanoparticles and large grains were metallic Ni and BZCY perovskite phase, respectively (Figure 1f,g). The nanoparticle and main phase interplanar spacings of 0.202 and 0.181 nm were assigned to metallic Ni (111) and BZCY (211) planes, respectively. Interestingly, the nanozone (Region 2) in Figure 1f did not exhibit a lattice structure, suggesting that it was amorphous BaO in combination with the EDX results.

The composition and morphology of the electrode fabricated by the impregnation and limited reaction method was also characterized (Figures S4–S7, Supporting Information). Again, the successful Ni doping into the perovskite lattice was confirmed by the decrease in the lattice parameter and the uniform distribution of Ba, Zr, Ce, Y, and Ni in BZCYN sample (Figures S4–S6, Supporting Information). The SDC scaffold displayed a highly porous architecture, built from well‐distributed particles with the size of 200–400 nm (Figure S7a, Supporting Information). After impregnating the porous SDC scaffold with BZCYN phase and then calcining it, its walls were decorated with many BZCYN nanoparticles with the size of 100 nm (Figure S7b, Supporting Information). After further treatment with H_2_, some even smaller nanoparticles with the size of ≈30 nm appeared on the surface of the impregnated perovskite phase, which were assigned to the metallic Ni phase (Figure S7c, Supporting Information). The phase structure and morphology of the thin porous layer on the SDC scaffold surface (Figure S8, Supporting Information) were similar to those of the reduced BZCYN powder (Figure 1d–g), suggesting that the compositional and morphological investigation of the reduced BZCYN powder could provide insight into the structures of the reduced BZCYN‐infiltrated SDC anode. Thus, the dark nanoparticles (≈30 nm in size), lighter particles, and amorphous region were assigned to Ni, BZCY, and BaO phases, respectively (Figure S8, Supporting Information).

Figure S9 in the Supporting Information shows that the limited reduction reaction, during which the Ni nanoparticles and amorphous BaO phase were formed, led to a simultaneous increase in the pore volume and Brunauer–Emmett–Teller (BET) surface area. More specifically, the BET surface area of BZCYN increased from 4.58 to 7.77 m^2^ g^−1^ after H_2_ reduction. The total pore volumes of the BZCYN and reduced BZCYN were 0.019 and 0.039 mL g^−1^, respectively. The porous nature of the reduced BZCYN layer might facilitate gas diffusion, and the enlargement in the surface area increased the triple phase boundary (TPB) length. Both of these changes are advantageous for fuel oxidation at the electrode.

**Figure** **2**a–f shows the 3D surface‐rendered image of the individual phases (SDC, BZCYN, and pores) in the BZCYN‐infiltrated SDC anode based on focused ion beam SEM (FIB‐SEM) images (Figure S10, Supporting Information). The individual phases were separated by image thresholding. The BZCYN dispersion in the infiltrated anodes was significantly enhanced after reduction, as shown by more numerous and smaller particles (Figure 2g,h). The enhanced catalyst dispersion due to the increased pore volume after H_2_ treatment should result in an increase in the number of active sites or the TPB length and thus enhance the electroactivity of the electrode.

**Figure 2:**
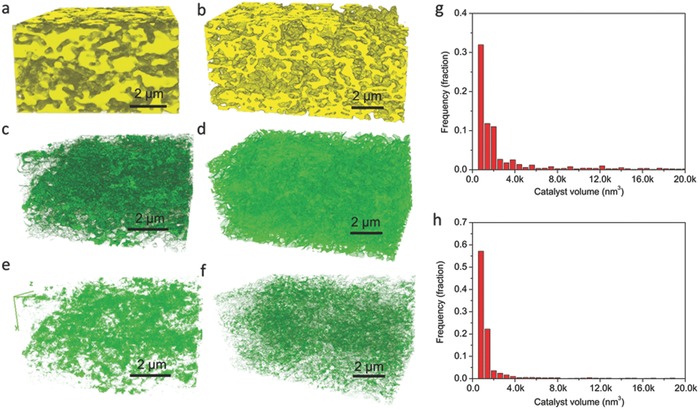
3D surface‐rendered image obtained from the segmented FIB‐SEM tomograms of the BZCYN‐infiltrated SDC anodes before (left) and after (middle) reduction in H_2_: a,b) SDC, c,d) BZCYN, e,f) voids. BZCYN particle size distributions (right) derived from image analysis of the FIB‐SEM tomogram g) before and h) after reduction in H_2_.

To demonstrate the potential of this unique electrode architecture as an SOFC anode, several cells with different anodes were fabricated, and their electrochemical performances were compared. A cell with reduced BZCYN anode delivered a peak power density (PPD) of 184 mW cm^−2^ at 800 °C operating on H_2_, which was 3.6 times that of a cell with Ni‐free BZCY anode, indicating the importance of the exsolved Ni phase for H_2_ electro‐oxidation reaction (Figure S11a, Supporting Information). Specifically, this phase improves both the electronic conductivity and catalytic activity of the anode. A cell consisting of reduced BZCYN‐infiltrated SDC anode, thick SDC electrolyte and Ba_0.5_Sr_0.5_Co_0.8_Fe_0.2_O_3−_ *_δ_* + SDC cathode (Figure S12, Supporting Information) with the thicknesses of ≈30, 300, and 20 µm, exhibited PPDs of 590, 535, 458, 369, and 297 mW cm^−2^ operating on H_2_ fuel at 800, 750, 700, 650, and 600 °C, respectively (Figure S11b, Supporting Information). These high power outputs are remarkable considering the thickness of the electrolyte (300 µm). The much higher power output of the cell with BZCYN‐infiltrated SDC anode compared with the cell with BZCYN anode indicates that the electrode microstructure significantly affects the H_2_ electro‐oxidation reaction. Furthermore, the use of the SDC scaffold effectively increased the TPB length by extending the reaction region to encompass most of the electrode. **Figure** **3**a shows that the PPDs of the cells with reduced BZCYN‐ and Ni‐infiltrated SDC anodes were 590 and 464 mW cm^−2^ operating on H_2_, respectively, indicating that H_2_ electro‐oxidation was promoted by the BZCY phase. When the fuel was 200 ppm H_2_S in H_2_, the PPDs and electrode polarization resistances of the cell with the reduced BZCYN‐infiltrated SDC anode were similar to those obtained with H_2_ fuel (Figure 3b; Figure S13, Supporting Information), demonstrating the excellent sulfur tolerance of this anode. Figure 3b shows that when the fuel was 200 ppm H_2_S in H_2_, the PPDs of the cells with the reduced BZCYN‐infiltrated SDC, Ni‐infiltrated SDC, and BZCY‐infiltrated Ni+SDC anodes were 561, 424, and 381 mW cm^−2^, respectively, which corresponds to decreases of 4.9%, 8.6%, and 13.2% relatively to those obtained with pure H_2_. These results demonstrated the superior sulfur tolerance of the reduced BZCYN‐infiltrated SDC anode, which was further confirmed by the differences in the electrode polarization resistances (Figure S14, Supporting Information). As shown in Figure S14 in the Supporting Information, it was found that there were some differences in the ohmic resistances, which could be attributed to the reconstruction, diffusion, and loss of the Ni active sites in the Ni‐infiltrated SDC and BZCY‐infiltrated Ni+SDC anodes.^23^, ^25^ Due to the strong water‐storage capability of amorphous BaO and BZCY proton conductor in the reduced BZCYN‐infiltrated SDC anode, the deposited sulfur on the anode surface can be rapidly eliminated, which can prevent the formation of nickel sulfides (Ni_2_S_3_) on the Ni surface. However, due to the poor sulfur tolerance of the Ni‐infiltrated SDC and BZCY‐infiltrated Ni+SDC anodes, the sulfur adsorbed on Ni surface cannot be eliminated immediately, resulting in the formation of Ni_2_S_3_, which was easily to be dissociated at 800 °C since its melting point was only 787 °C.^5^ As a result, the dissolution of Ni_2_S_3_ led to the continuous reconstruction, diffusion, and loss of Ni in the anode, which may cause the large differences in the ohmic resistances of the cells with different infiltrated anodes.

**Figure 3:**
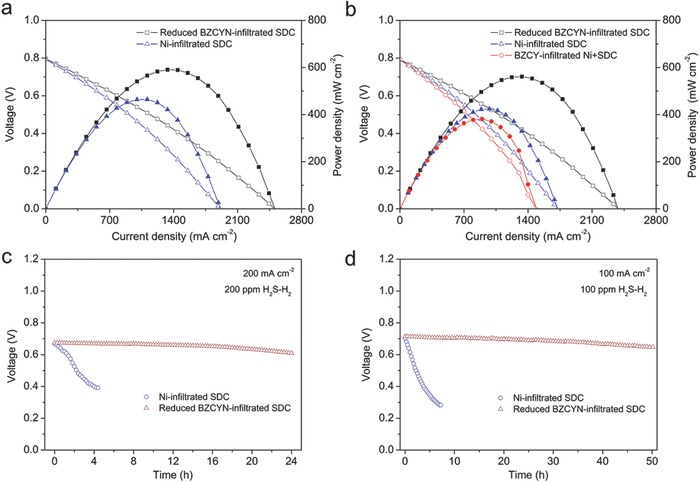
*I–V* curves for SOFCs with different infiltrated anodes obtained using a) H_2_ and b) 200 ppm H_2_S in H_2_ at 800 °C. Stability tests for SOFCs with reduced BZCYN‐ and Ni‐infiltrated SDC anodes obtained with c) 200 and d) 100 ppm H_2_S in H_2_ at 800 °C.

In addition to the power density, the operational stability of the electrode is also critical for practical applications. Figure 3c,d shows that the voltage of the cell with the reduced BZCYN‐infiltrated SDC anode remained nearly constant at 0.7 V for 24 and 50 h in 200 and 100 ppm H_2_S–H_2_ fuels, respectively. In contrast, the voltages of the cells with Ni‐infiltrated SDC anode rapidly decreased with time on stream. For practical application, the fuel cell may be required multiple start‐up and shut‐down operations in its whole lifetime, thus a high thermal stability and thermomechanical compatibility of the cell is required. The cell was heated up to 700 °C in H_2_ at a rate of 10 °C min^−1^ and holding for 20 min to achieve stable open‐circuit voltage (OCV) and PPD, and then the cell was cooled down to 200 °C in H_2_ at a rate of 2 °C min^−1^. The effect of thermal cycling on the anode stability was monitored by the power output of the fuel cell as shown in Figure S15 in the Supporting Information. The OCV and PPD of the single cell were well maintained at 0.88 V and 360 mW cm^−2^ in the 14 cycles involving the quick heating‐up and cooling‐down procedures, suggesting the superior thermal cyclability of the reduced BZCYN‐infiltrated SDC anode.

For the infiltrated anodes, the thermal expansion behavior is very close to the electrolyte, which ensured the long‐term stability of SOFCs. As shown in Figure S16 in the Supporting Information, the thermal expansion coefficient (TEC) value of the SDC scaffold was 12.4 × 10^−6^ K^−1^, which agrees well with that reported in the literature.^26^ A little lower TEC value of 11.4 × 10^−6^ K^−1^ was obtained for the reduced BZCYN‐infiltrated SDC anode in comparison with the fresh one (12.6 × 10^−6^ K^−1^), which could be attributed to the formation of Ni nanoparticles and amorphous BaO in the reduction process. It suggested the reduced BZCYN‐infiltrated SDC anode was highly compatible with SDC electrolyte, such good thermomechanical compatibility ensures a good operational stability.

It is well known that the electrode reaction usually appears at the region of TPB where the electrolyte (oxygen‐ion‐conducting phase), the electrode (electronic‐conducting phase), and the gas phase meet. An increase in the TPB length will provide larger number of active reaction sites, thus an improvement in electrode performance is expected. Because of the high ionic conductivity of SDC, the oxygen‐conducting phase was successfully penetrated into the electrode layer by applying the SDC scaffold. As a result, the TPB length is effectively increased by extending the reaction region into the bulk of electrode. Besides, the formation of porous structure from the limited conversion reaction exposed more active sites to surrounding atmosphere, thus the TPB length was further increased. It indicates that the building of the special hierarchical porous architecture through impregnation and limited conversion reaction greatly increased the TPB length, thus contributing to the superior electrocatalytic activity for H_2_ oxidation. In addition to the TPB length, the electrochemical activity of an electrode for fuel oxidation is also closely related to its intrinsic activity. It is well demonstrated that the reduction of particle size to the nanometer range can introduce some unusual properties, such as boosting the catalytic activity. In particular, recently, it was demonstrated that nickel nanoparticles showed outstanding performance for fuel electro‐oxidation.^27^, ^28^, ^29^ Clearly, the creation of abundant nickel nanoparticles from the limited conversion reaction of BZCYN further improved the electrode performance for H_2_ electro‐oxidation for power generation.

Typically, nickel is easily poisoned by sulfur due to the easy adsorption of sulfur on the nickel surface for the low H_2_S dissociation energy. The sulfur adsorption over the nickel surface will block the catalytic reaction for fuel electro‐oxidation. As a result, a quick deterioration for the conventional nickel‐based electrode performance may be experienced when a small amount of sulfur was presented in the fuel gas. Sulfur poisoning can be reduced by two different strategies: changing the Ni electronic structure or increasing the rate of sulfur removal from the Ni surface. The electronic structure of Ni can be altered by alloying or changing the interaction between Ni and the substrate. As shown in Figure S17 in the Supporting Information, the onset temperature and peak temperature are two most important parameters to embody the easiness of the reduction of NiO, which are closely related to the NiO‐substrate interaction. The NiO was reduced at an onset temperature of around 203 °C, and the peak temperatures were 260 and 351 °C, which matched pretty well with the literature.^22^ For the NiO+SDC anode, the onset temperature and the peak temperature were found to be 280 and 409 °C, respectively, which were comparable to those of the free NiO, respectively, suggesting the weak interaction between NiO and SDC in the conventional NiO+SDC anode. As to the BZCYN‐infiltrated SDC, a reduction onset temperature of 300 °C and peak temperatures of 396 and 615 °C were derived from the hydrogen temperature‐programmed reduction (H_2_‐TPR) profiles, suggesting the BZCYN‐infiltrated SDC anode showed stronger Ni‐substrate interaction compared to the NiO+SDC anode, which could contribute to its superior sulfur tolerance by reducing sulfur adsorption (Figure S17, Supporting Information). In addition, sulfur poisoning can be reduced by gasifying the deposited/adsorbed sulfur on the anode. The reduced BZCYN anode could store more water than Ni+SDC anode due to the presence of BZCY and amorphous BaO phases (Figure S18, Supporting Information). A water desorption peak was observed from an onset temperature of 55 °C and peaked at 107 and 293 °C, suggesting the BZCYN was water storable. This is well understood since both BZCYN and BZCY are proton conductors, and the amorphous BaO also contributed to the water storage. For comparison, the conventional Ni+SDC anode showed almost no water‐storage capability. The superior sulfur tolerance of the reduced BZCYN‐infiltrated SDC anode was probably due to both the enhanced Ni‐substrate interaction and higher water‐storage capacity.

Based on the above analysis, the superior performance of the SOFC anode modified with amorphous BaO and Ni nanoparticles could be explained by the mechanism shown in **Figure** **4**. First, H_2_ fuel is oxidized from the cathode at TPB to generate water (Equation (1)), and sulfur simultaneously adsorbed on Ni surface to form a surface‐adsorbed sulfur species $\left(S_{\mathrm{Ni}}^{*}\right)$ (Equation (2)). Water is subsequently stored in BZCY by (OH)_o_ formation (Equation (3)). Meanwhile, water is also physically adsorbed on amorphous BaO surface (Equation (4)). Then, the (OH)_o_ species and physically adsorbed water can react with $\left(S_{\mathrm{Ni}}^{*}\right)$ to generate SO_2_ and H_2_. Finally, SO_2_ desorbs from the Ni surface (Equations (5) and (6)), whereas H_2_ is oxidized to form H_2_O

(1) $$H_{2}+O^{2-}\;\to \;H_{2}O+2e^{-}$$

(2) $$H_{2}S+\mathrm{Ni}\to S_{\mathrm{Ni}}^{*}+H_{2}$$

(3) $$H_{2}O+O_{o}^{\times }+V_{o}^{••}\to 2{\left(\mathrm{OH}\right)}_{o}^{•}$$

(4) $$\mathrm{BaO}\;+\;H_{2}O\;\to \;{\mathrm{Ads}}_{H_{2}O}\;\mathrm{on}\;\mathrm{BaO}$$

(5) $$S_{\mathrm{Ni}}^{*}+\;2{\left(\mathrm{OH}\right)}_{o}^{•}+2e^{–}\to {\mathrm{SO}}_{2}+H_{2}$$

(6) $$S_{\mathrm{Ni}}^{*}\;+\;2{\mathrm{Ads}}_{H_{2}O}\;\mathrm{on}\;\mathrm{BaO}\;\to \;{\mathrm{SO}}_{2}\;+\;2H_{2}$$

**Figure 4:**
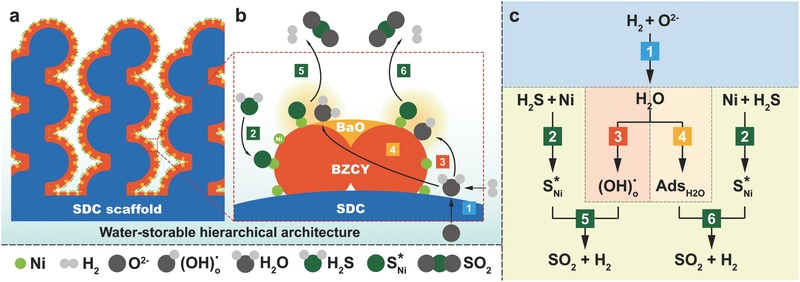
Proposed mechanism for water‐induced sulfur removal from the hierarchically structured anode modified with Ni nanoparticles and amorphous BaO.

In summary, a highly active, sulfur‐tolerant, water‐storable anode was successfully fabricated by impregnation, followed by calcination and a limited reaction. This anode had a hierarchical structure that was modified with amorphous BaO and Ni nanoparticles. It exhibited excellent chemical and thermal compatibility with the other cell components, good sulfur tolerance, high electro‐oxidation activity, and excellent thermomechanical stability. The cell constructed with this anode exhibited better stability in the H_2_S–H_2_ fuel than that with a conventional Ni+SDC anode, due to the stronger Ni‐substrate interaction and higher water‐storage capacity. This work demonstrated a novel, effective approach for developing sulfur‐tolerant SOFC anodes, which can accelerate the commercialization of SOFC technology.

## Experimental Section

Experimental details are included in the Supporting Information.

## Conflict of Interest

The authors declare no conflict of interest.

## Supporting information

Supplementary
