# Supplementary material for: Rational Design of a Water‐Storable Hierarchical Architecture Decorated with Amorphous Barium Oxide and Nickel Nanoparticles as a Solid Oxide Fuel Cell Anode with Excellent Sulfur Tolerance
Source: Adv Sci (Weinh). 2017 Sep 15;4(11):1700337. doi: 10.1002/advs.201700337 (PMC5700654; doi:10.1002/advs.201700337)
Supplement: Supplementary file 1 — Supplementary [file ADVS-4-na-s001.pdf]

Copyright WILEY-VCH Verlag GmbH & Co. KGaA, 69469 Weinheim, Germany, 2017.

ADVANCED SCIENCE
Open Access

Supporting Information

for *Adv. Sci.,* DOI: 10.1002/advs.201700337

Rational Design of a Water-Storable Hierarchical Architecture Decorated with Amorphous Barium Oxide and Nickel Nanoparticles as a Solid Oxide Fuel Cell Anode with Excellent Sulfur Tolerance

Yufei Song, Wei Wang, Lei Ge, Xiaomin Xu, Zhenbao Zhang, Paulo Sérgio Barros Julião, Wei Zhou, and Zongping Shao**

Copyright WILEY-VCH Verlag GmbH & Co. KGaA, 69469 Weinheim, Germany, 2017.

Supporting Information

Rational Design of a Water-Storable Hierarchical Architecture Decorated with Amorphous Barium Oxide and Nickel Nanoparticles as a Solid Oxide Fuel Cell Anode with Excellent Sulfur Tolerance

Yufei Song, Wei Wang, Lei Ge, Xiaomin Xu, Zhenbao Zhang, Paulo Sérgio Barros Julião, Wei Zhou, and Zongping Shao**

Y. Song, Dr. Z. Zhang, Prof. W. Zhou
State Key Laboratory of Materials-Oriented Chemical Engineering, College of Chemical Engineering, Jiangsu National Synergetic Innovation Center for Advanced Materials (SICAM), Nanjing Tech University, No. 5 Xin Mofan Road, Nanjing 210009, P.R. China
E-mail: zhouwei1982@njtech.edu.cn

Dr. W. Wang, X. Xu, P. S. B. Julião, Prof. Z. P. Shao
Department of Chemical Engineering, Curtin University, Perth, Western Australia 6845, Australia

Dr. L. Ge
Center for Future Materials, University of Southern Queensland, Springfield Central, Queensland 4300, Australia

Prof. Z. P. Shao
State Key Laboratory of Materials-Oriented Chemical Engineering,
School of Energy Science and Engineering, Jiangsu National Synergetic Innovation Center for Advanced Materials (SICAM), Nanjing Tech University, No. 5 Xin Mofan Road, Nanjing 210009, P.R. China
E-mail: shaozp@njtech.edu.cn

1. Experimental Section

Materials preparation: $BaZr_{0.4}Ce_{0.4}Y_{0.2}O_{3-\delta}$ (BZCY), $Ba(Zr_{0.4}Ce_{0.4}Y_{0.2})_{0.8}Ni_{0.2}O_{3-\delta}$ (BZCYN), $Sm_{0.2}Ce_{0.8}O_{1.9}$ (SDC), $Ba_{0.5}Sr_{0.5}Co_{0.8}Fe_{0.2}O_{3-\delta}$ (BSCF) and $Sm_{0.5}Sr_{0.5}CoO_{3-\delta}$ (SSC) composite oxide powders, that were used in this study, were prepared via a combined EDTA-citrate complexing method with the detailed synthesis process described elsewhere. [1] Phase-pure samples were obtained by calcination of their solid precursors at 1000, 1000, 800, 950 and 1000 ^oC in air for 5 h, respectively. The BZCYN perovskite was reduced in H_2 at 800 ^oC for 10 h to obtain Ni nanoparticles and amorphous BaO co-decorated BZCY anode. Aqueous nitrate solutions of BZCY and BZCYN precursors for infiltration were prepared by dissolving stoichiometric amounts of $Ba(NO_3)_2$, $Zr(NO_3)_4{\cdot}5H_2O$, $Ce(NO_3)_3{\cdot}6H_2O$, $Y(NO_3)_3{\cdot}6H_2O$ and $Ni(NO_3)_2{\cdot}6H_2O$ in deionized water with a concentration of 0.2 mol L^{-1}. Appropriate amount of citric acid was also added to the solution as a complexing agent to facilitate the formation of the desired perovskite phase at relatively low temperatures.

Cell fabrication: The single cells were prepared as follows. First, under the hydraulic pressure of 200 MPa, the as-synthesized SDC powder was pressed into disk-shaped pellets. These SDC pellets were sintered at 1400 ^oC in air for 5 h, and then their surfaces were polished with sand paper to reach 300 μm in thickness. The appropriate SDC powder and 10 wt.% soluble starch (pore former) were dispersed in ispropyl alcohol to form a suspension that was sprayed onto both surfaces of the as-fabricated SDC electrolyte with an effective surface area of 0.45 cm^{-2}. The composite was fired at 1250 ^oC in air for 5 h to yield a porous SDC scaffold with a thickness of ~30 μm. Finally, the as-prepared BZCY or BZCYN precursor solutions were infiltrated into the SDC scaffold, after each infiltration, the pellet was calcined in air at 400 ^oC for 30 min for the decomposition of the metal nitrates and citric acid. Multiple steps were required to reach the final loading of ~25 wt.%. Afterwards, the composite anode was calcined in air at 1000 ^oC for 5 h. A similar process was also used to prepare the Ni-infiltrated SDC anode and BZCY-infiltrated Ni+SDC (6:4, weight ratio) anode for the comparison

purpose. In these four anodes, the Ni amount was all well controlled at 5 wt.%, the same to the Ni amount in the reduced BZCYN-infiltrated SDC anode. Silver ink (current collector) was applied to both electrode surfaces and silver wires were selected as the current leads.

Electrochemical measurements: The *I-V* and *I-P* curves of the fuel cells were obtained using a Keithley 2420 source meter based on a four-probe configuration. During the measurements, H_2, 100 ppm H_2S-H_2 or 200 ppm H_2S-H_2 fuels were fed into the anode chamber and ambient air was used as the cathode atmosphere. The flow rate of H_2, 100 ppm H_2S-H_2 or 200 ppm H_2S-H_2 fuel was kept constant at 80 mL min^{-1} [STP]. The single cells were assessed over the temperature range of 600-800 ^oC. The impedance of the fuel cell was investigated by the electrochemical impedance spectroscopy (EIS) measurement using a Solartron 1260 frequency response analyzer in combination with a Solartron 1287 potentiostat, performed at open circuit voltage (OCV) condition. The frequency of the EIS measurement ranged from 0.1 to 1000 kHz and the signal amplitude was 20 mV. To avoid potential CO_2 poisoning and phase transition of the BSCF cathode, SSC with a lower activity for oxygen reduction reaction (ORR) yet better stability was used as the cathode for the stability test. To exclude the negative effect of potential degradation of the Ag paste current collector on the operational stability, a mesh-like Ag was applied as the current collector during the stability evaluation.[2]

Materials characterizations: The phase structure of the prepared samples was examined by X-ray diffraction (XRD, D8 Advance Bruker) using a Cu-Kα radiation. The data was collected in a step-scan mode within a 2-theta range of 20-80° with intervals of 0.02^o. The microstructure of the BZCYN-infiltrated electrodes was characterized by a field emission scanning electron microscope (FE-SEM, JEOL-S4800) and high resolution-transmission electron microscope (HR-TEM, JEOL JEM-2100). Bright-field scanning transmission electron microscopy (STEM) images were obtained using an FEI Tecnai G2 T20 electron microscope operating at 200 kV. The corresponding energy-dispersive X-ray (EDX) mappings were acquired using a TEM equipped with an EDX analyzer operating at 300 kV

(FEI Tecnai G2 F30 STWIN). The thermal expansion coefficient (TEC) data was collected using a Netzsch DIL 402C/3/G dilatometer in Ar from 200 to 800 ^{o}C with a heating rate of 5 $^{o}C\ min^{-1}$. X-ray photoelectron spectra (XPS) measurements were performed on a Thermo ESCALAB 250 using monochromatic Al Kα radiation (1486.6 eV). Focused ion beam scanning electron microscopy (FIB-SEM) was performed in a FEI SCIOS FIB/SEM dual beam system to assess the contact of the inorganic phase and the continuous phase. The specimen was sputtered with a conducting layer of Pt for 100 s. A trench was milled on the surface of the membrane by using a Ga^{+} focused ion beam. Serial milling of slices with a thickness of 80 nm were removed from the specimen up to a depth of 30 μm by the Ga^{+} FIB at 30 kV and 3-4 nA. A series of exposed cross-section SEM images in back-scattered electron (BSE) imaging mode were collected sequentially during the automatic slice-and-view experiments using an in-lens back-scattered electron detector at 2 kV. In the BSE SEM image, the different constituents of the anode can be recognized through the different grayscales. SDC is the brightest whereas BZCYN appears as a medium grayscale and pores are the darkest. The segmentation of the individual phases (e.g., SDC, BZCYN and pore) was conducted by image thresholding. The stack of these SEM images was aligned, and the analyzed volume can be reconstructed in three dimensions (3D). Avizo (FEI Visualization Sciences Group) was used to reconstruct the tomograms, segment different phases and quantify the corresponding 3D volume. The pore structure and Brunauer-Emmett-Teller (BET) specific surface areas of the BZCYN before and after hydrogen reduction was analyzed by nitrogen adsorption-desorption isotherms from a Quantachrome AutoSorb-iQ3 instrument at the liquid nitrogen temperature. The water storing capability of reduced BZCYN was evaluated by mass spectrometry (MS) in a mass spectrometer (QMS 403D Aëolos®, NETZSCH) that was combined with a thermogravimetric analyzer (STA 449 F3 Jupiter®, NETZSCH). In hydrogen temperature-programmed reduction (H_2-TPR), approximately 0.03 g of the samples was put in a U-type quartz reactor with an inner diameter of about 3 mm.

The sample was pretreated under a pure argon atmosphere at a flow rate of 30 ml min^{-1} [STP] at 400 °C for 30 min. After cooling to room temperature, the atmosphere was switched to 10 vol.% H_2-Ar, and the reactor was programmatically heated to 800 °C at a rate of 10 °C min^{-1}. The hydrogen consumption was monitored by an in situ thermal conductivity detector (TCD) with a BELCAT-A apparatus. BZCYN before and after the hydrogen treatment with the mass of 0.1 g was soaked in 25 mL deionized water for 10 h, and the water from the treatment was analyzed by inductively coupled plasma-atomic emission spectroscopy (ICP-AES, Optima 7000 DV, Perkin-Elmer, USA) to investigate the Ba^{2+} concentrations.

2. Supplementary Results

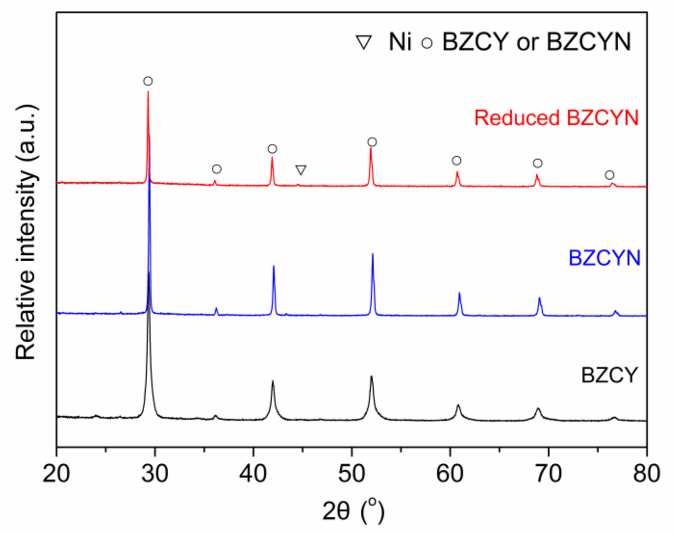

Figure S1. XRD patterns of BZCY, BZCYN and reduced BZCYN samples.

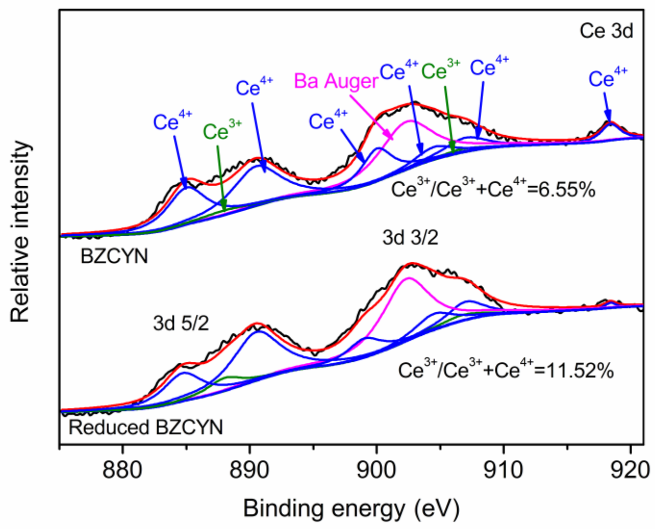

Figure S2. Ce 3d XPS spectra of the BZCYN and reduced BZCYN anodes.

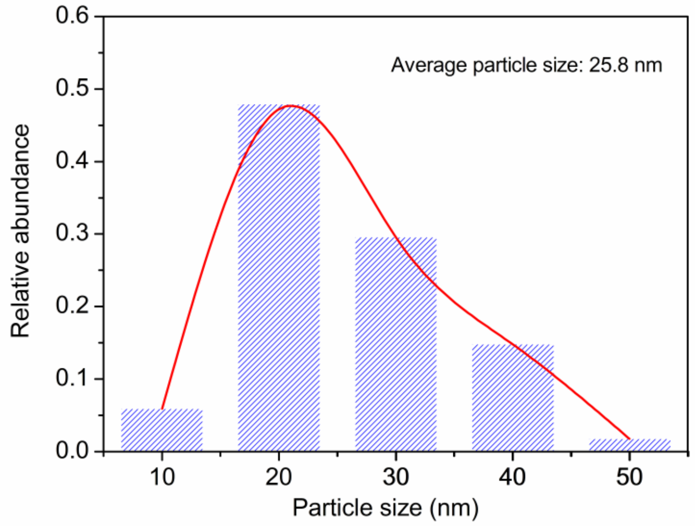

Figure S3. Particle size distributions of the Ni nanoparticles on BZCY surface based on the SEM results.

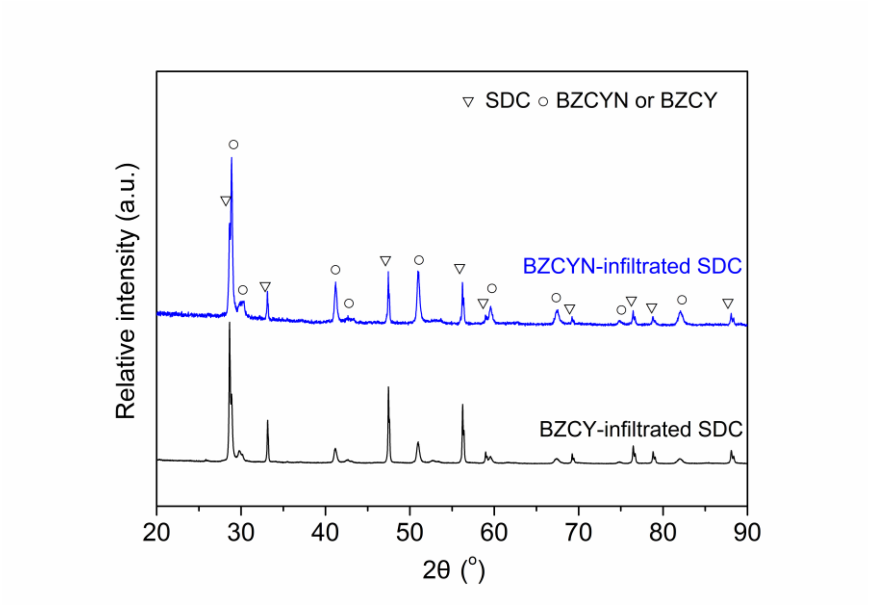

Figure S4. XRD patterns of BZCYN-infiltrated SDC and BZCY-infiltrated SDC anodes.

The BZCYN phase in BZCYN-infiltrated SDC anode displayed a smaller lattice parameter than that of the BZCY phase in BZCY-infiltrated SDC anode (4.380 vs. 4.382 Å), suggesting the successful Ni doping in BZCY perovskite lattice.

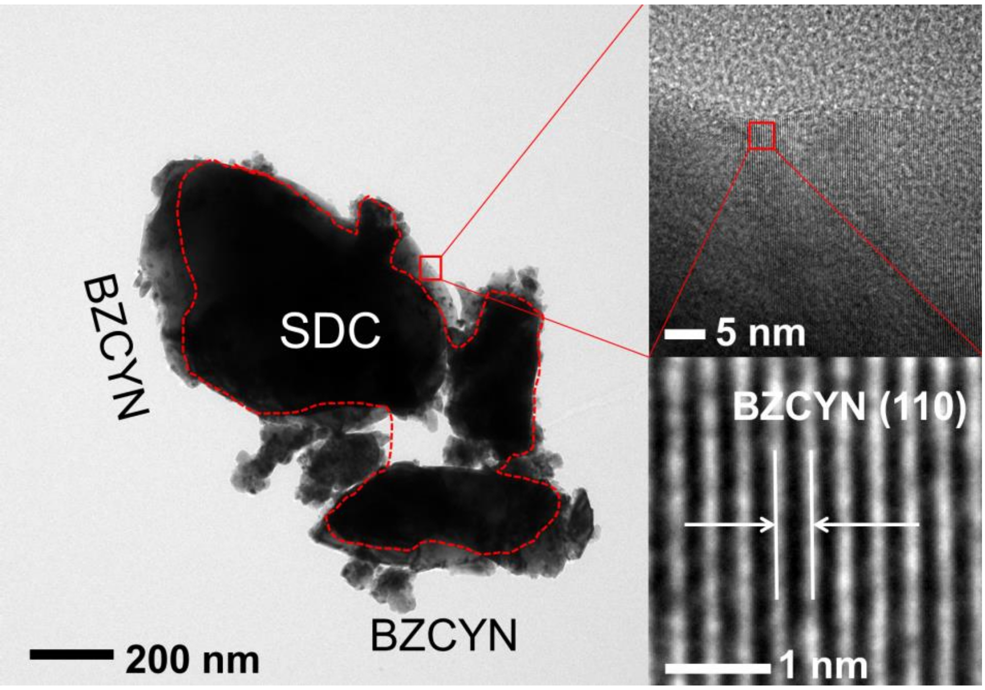

Figure S5. TEM images of the BZCYN-infiltrated SDC anode with different magnifications.

The interplanar spacing of 0.320 nm was assigned to BZCYN (110) plane.

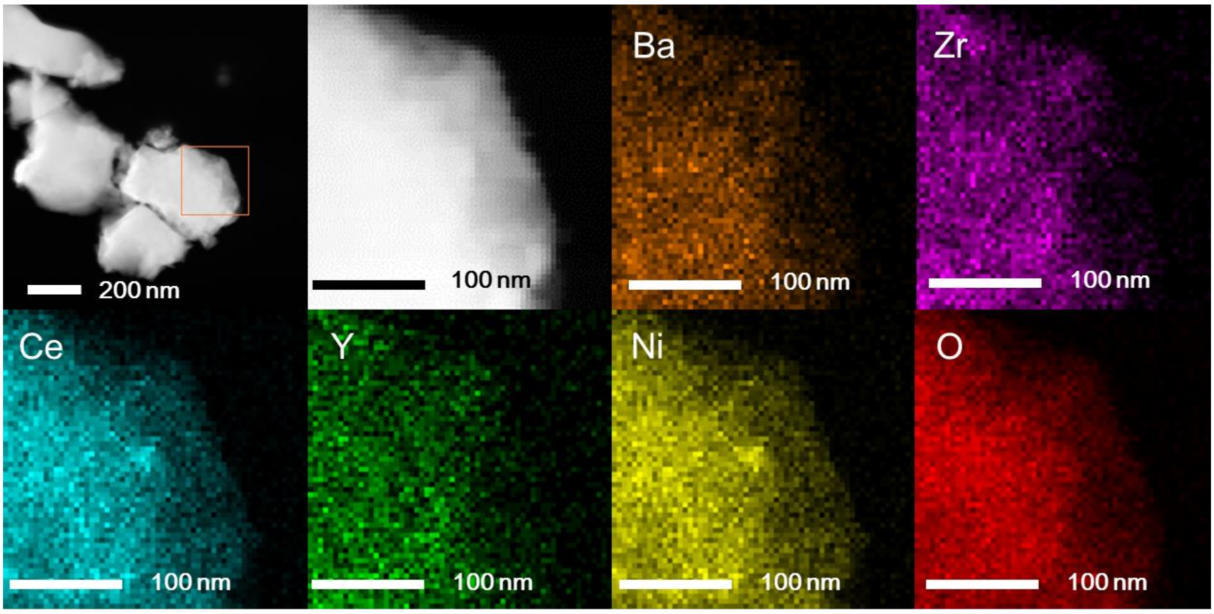

Figure S6. STEM-EDX profiles of the BZCYN-infiltrated SDC anode.

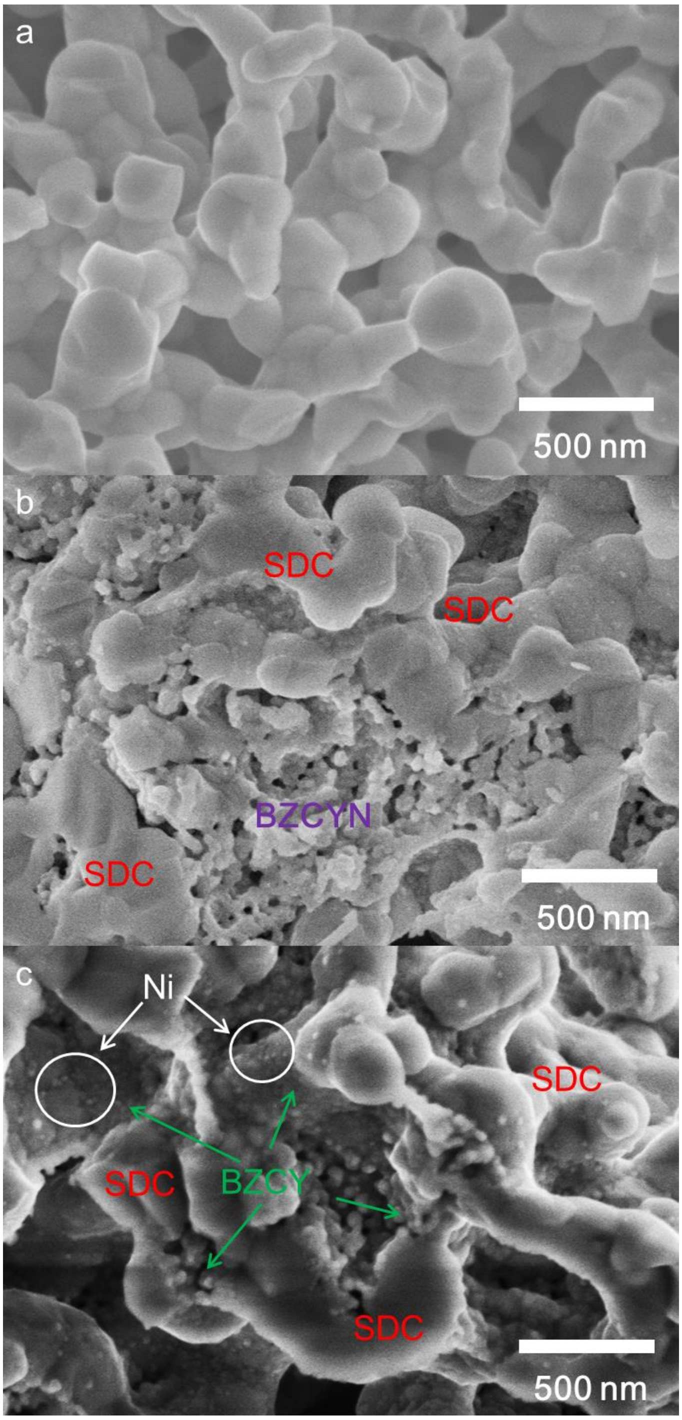

Figure S7. SEM images of (a) SDC scaffold, BZCYN-infiltrated SDC anodes before (b) and after reduction (c).

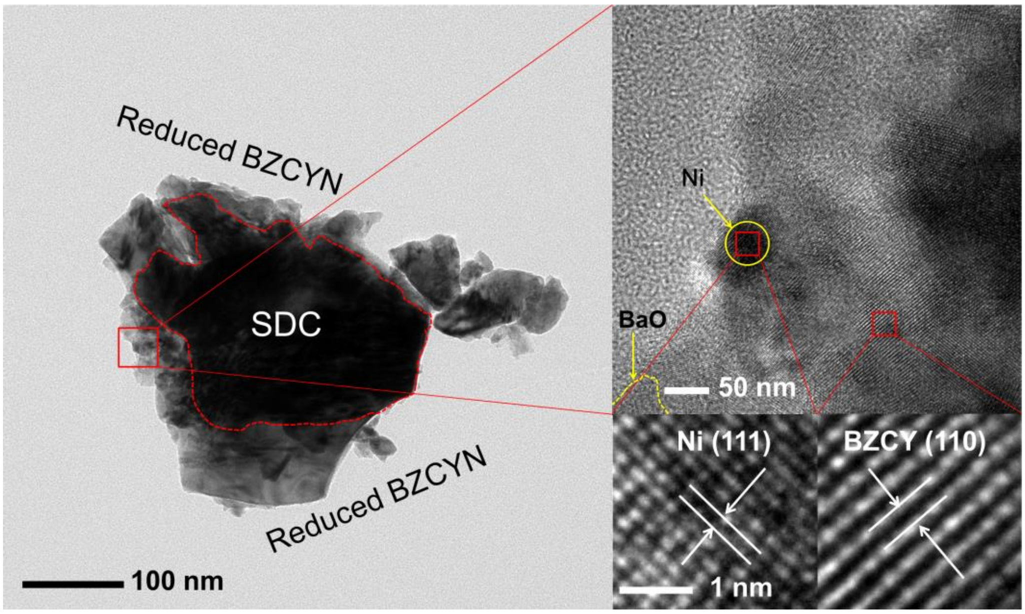

Figure S8. TEM images of the reduced BZCYN-infiltrated SDC anode.

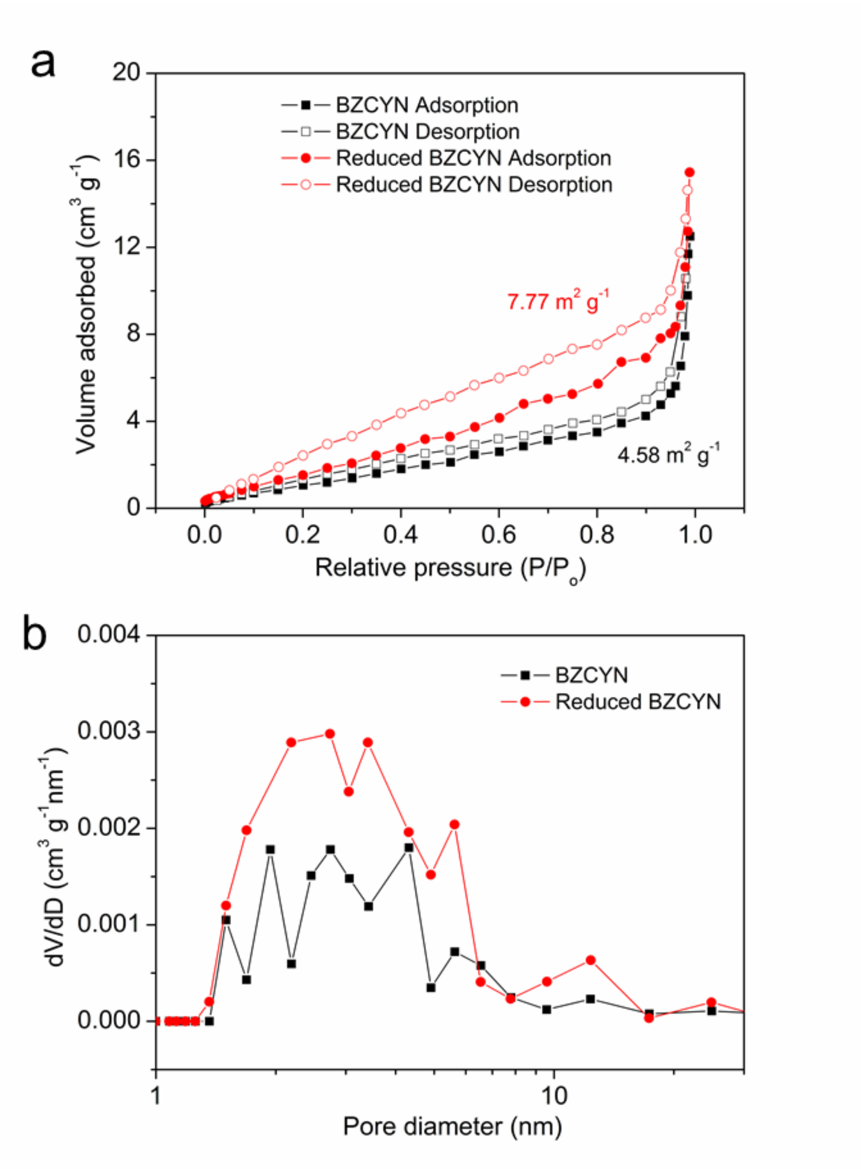

Figure S9. Nitrogen adsorption/desorption isotherms (a) and pore size distributions (b) of the BZCYN anodes before and after reduction in H_2 at 800 ^oC for 10 h.

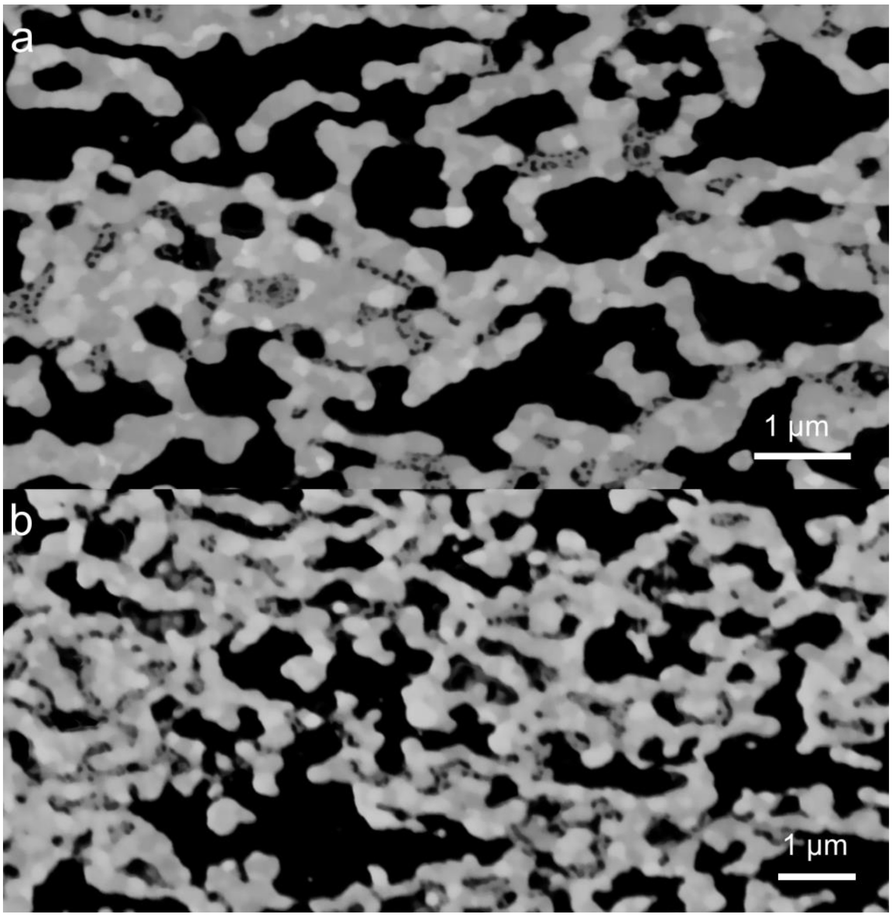

Figure S10. 2D FIB-SEM images of BZCYN-infiltrated SDC anodes before (a) and after (b) reduction in H_2 at 800 ^{o}C for 10 h. black: pore, white: SDC, grey: BZCYN

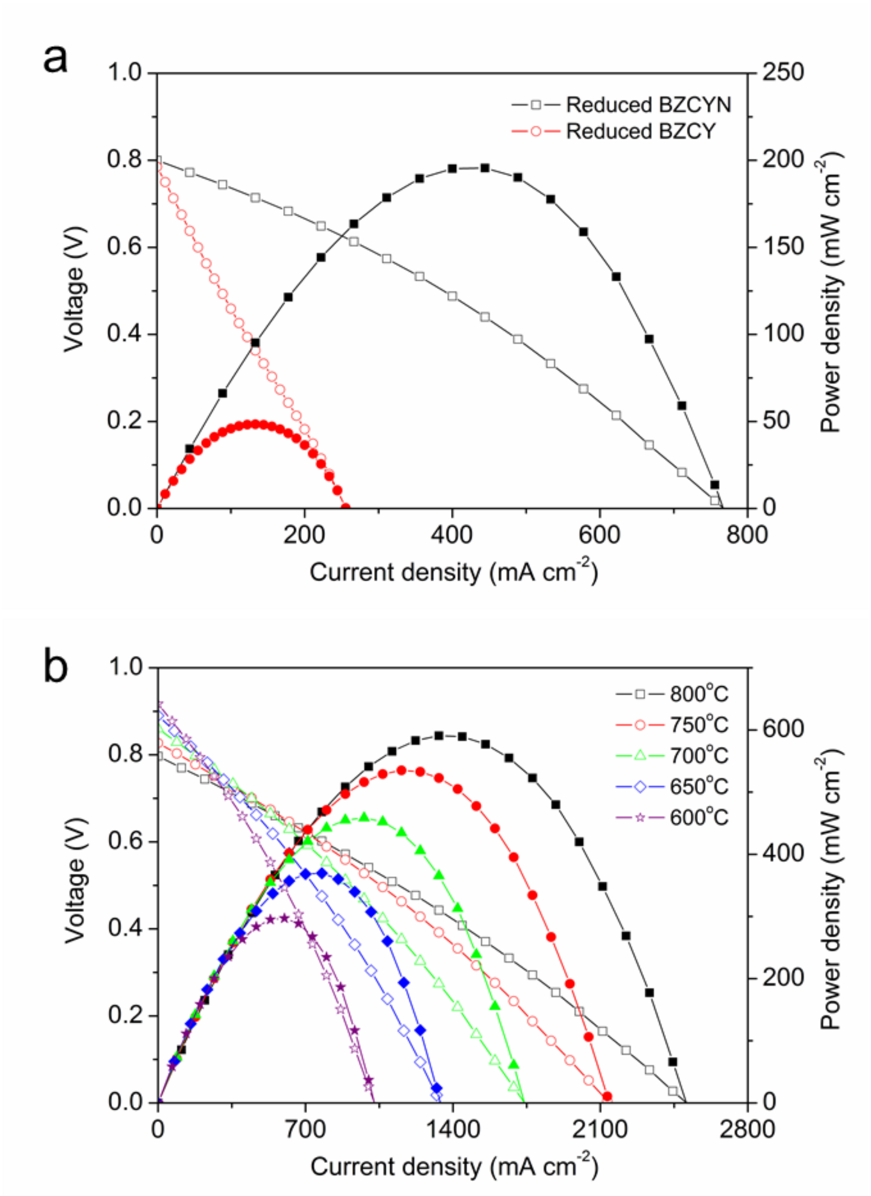

Figure S11. (a) *I-V* and *I-P* curves of the fuel cells with reduced BZCYN and BZCY anodes operating on H_2 at 800 °C. (b) *I-V* and *I-P* curves of the fuel cells with the reduced BZCYN infiltrated SDC anode operating on H_2 at 600-800 °C.

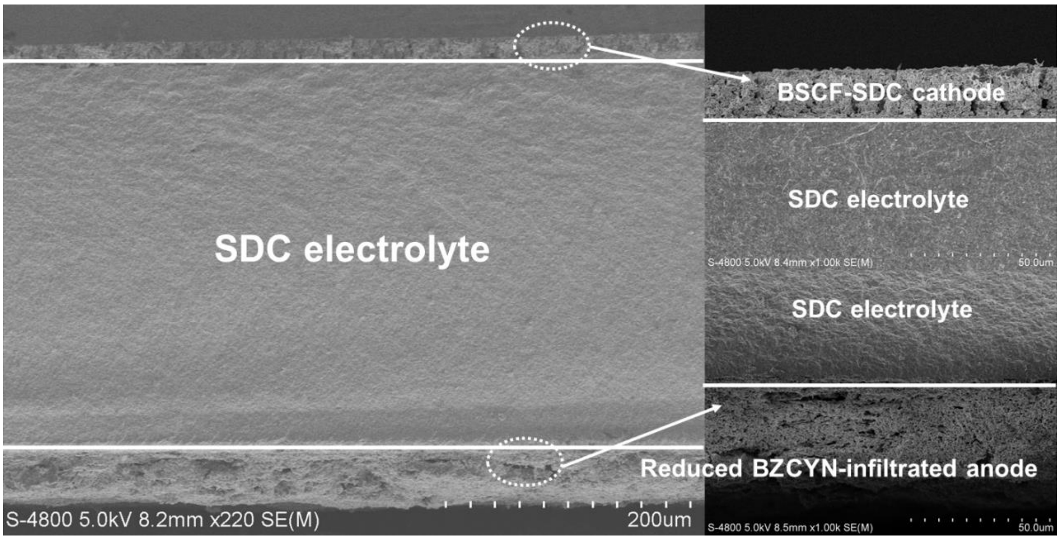

Figure S12. Typical SEM images of a cross-section of a fuel cell with reduced BZCYN-infiltrated SDC anode.

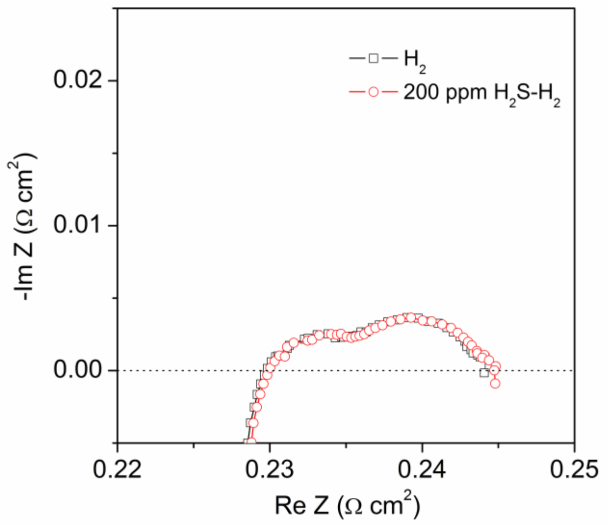

Figure S13. EIS spectra of the fuel cell with reduced BZCYN-infiltrated SDC anode operating on H_2 and 200 ppm H_2S-H_2 fuels at 800 ^oC.

The area-specific resistances (ASRs) of the fuel cells were determined from the impedance spectra. In EIS, the high-frequency offset on the real axis is primarily a result of the resistance of the electrolyte, whereas the difference between the high and low frequency intercepts on the real axis is associated with the electrode contribution, which includes both the anode and the cathode. Given the fact that the same cell was used operating on various fuels, those differences regarding electrode polarization resistances should be attributed solely to the anode side.

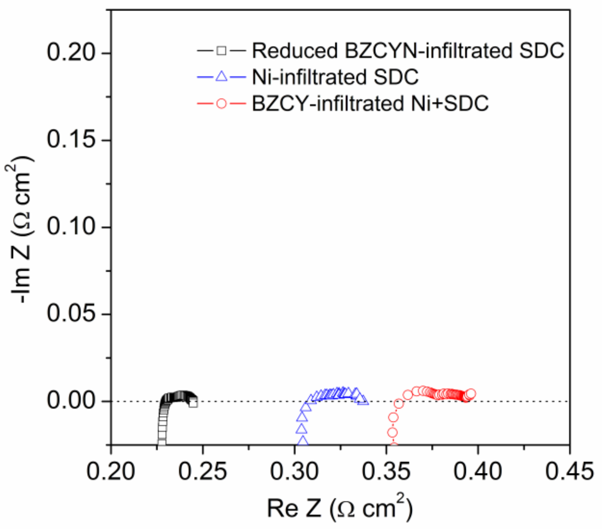

Figure S14. EIS spectra of the fuel cells with reduced BZCYN-infiltrated SDC, Ni-infiltrated SDC and BZCY-infiltrated Ni+SDC anodes operated on 200 ppm H_2S-H_2 at 800 °C.

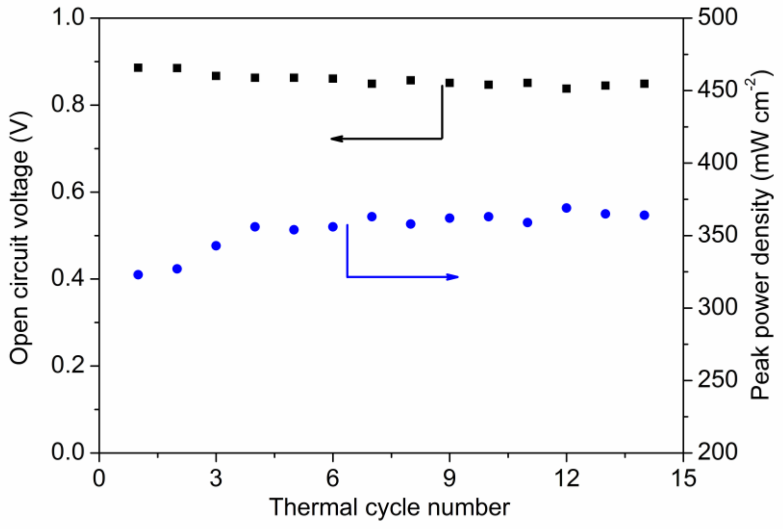

Figure S15. The thermal cyclability of a single cell with the reduced BZCYN-infiltrated SDC anode.

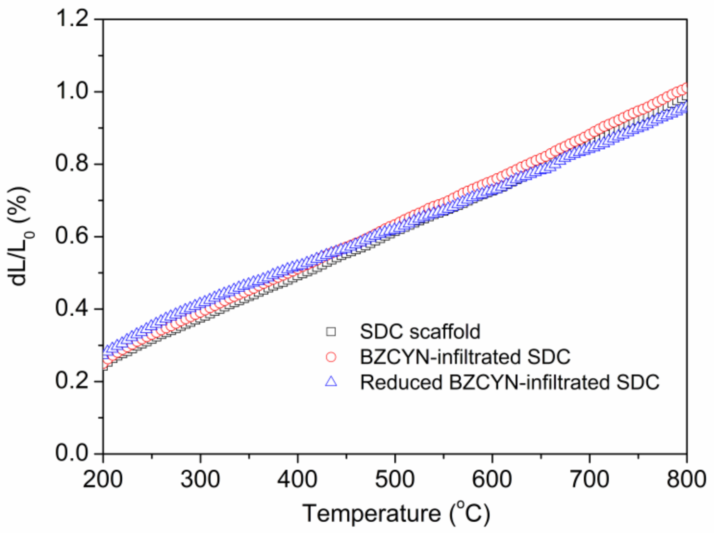

Figure S16. TEC curves of the SDC scaffold, BZCYN-infiltrated SDC and reduced BZCYN-infiltrated SDC anodes in Ar atmosphere.

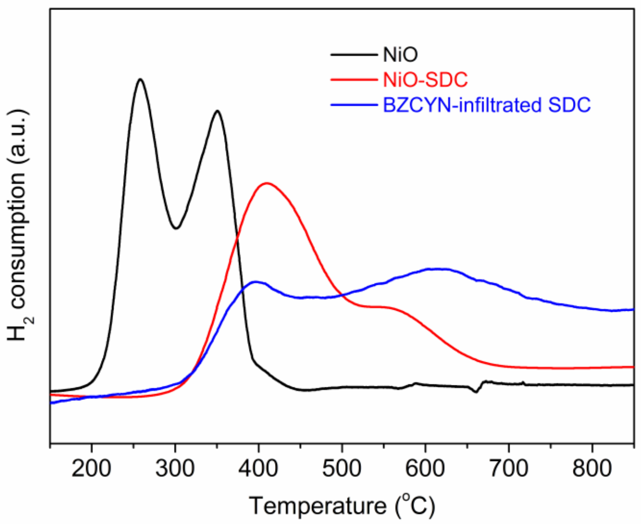

Figure S17. H_2-TPR profiles of the BZCYN-infiltrated SDC, NiO+SDC anodes and NiO.

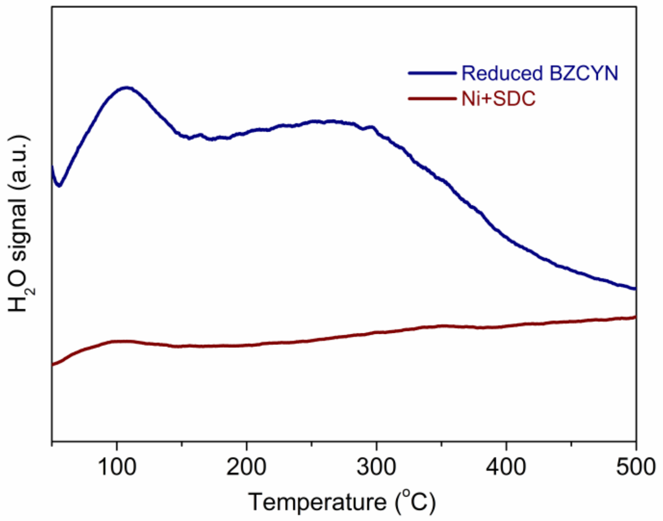

Figure S18. MS curves of reduced BZCYN-infiltrated SDC and Ni+SDC anodes.

References

[1] H. Gu, R. Ran, W. Zhou, Z. Shao, *J. Power Sources* **2007**, *172*, 704.
[2] Y. Chen, F. Wang, D. Chen, F. Dong, H. Park, C. Kwak, Z. Shao, *J. Power Sources* **2012**, *210*, 146.
